# New Appliances and Things Medical

**Published:** 1901-10-19

**Authors:** 


### NEW APPLIANCES AND THINGS MEDICAL.

**We shall be glad to receive, at our Office, 28 & 29 Southampton Street, Strand, London, W.C., from the manufacturers, specimens of all new preparations and appliances which may be brought out from time to time.]**

### CAMBBIDGE LEMONADE.

(Messrs. Chivers and Sons, Limited, Histon, Cambridge.)

Cambridge lemonade is a crystalline preparation which when dissolved in water provides a very agreeable and palatable beverage. Our examination of this preparation shows that it contains the organic acids and salts which are present in lemon juice, and the characteristic flavour of lemons due to the essential oil of lemons gives the beverage the same flavour which renders the home made varieties of lemonade so popular with the majority of people. We regard Cambridge lemonade as a very pure preparation. It is also very economical in use, and can be made at a moment's notice.

### PALM'S PATENT MALT EXTRACT BEER.

(Löwen Brauerei, Hamburg. London Agent: W. P. Goodall, 64 Leadenhall Street, London, E.C.)

This malt beverage is a highly concentrated non-intoxicating beverage for invalids. It contains something like 26 per cent. of malt extract and about 3 per cent. of alcohol. Its chemical composition renders this patent malt extract beer a valuable tonic for invalids, and moreover it is very pleasant to the palate; although to some degree stimulating its chief merit lies in its nutritive character, which makes this malt preparation one of considerable value for debilitated individuals and growing children.

